# Cysteine Dioxygenase Type 1 Inhibits Osteogenesis by Regulating Wnt Signaling in Primary Mouse Bone Marrow Stromal Cells

**DOI:** 10.1038/srep19296

**Published:** 2016-01-14

**Authors:** Xuefeng Zhao, Peng Deng, Jie Feng, Zheng Wang, Zichao Xiang, Xianglong Han, Ding Bai, Eung-Kwon Pae

**Affiliations:** 1 State Key Laboratory of Oral Diseases, West China Hospital of Stomatology, Sichuan University, China; 2 Department of Orthodontics, West China Hospital of Stomatology, Sichuan University, China; 3 Department of Orthodontics and Pediatric Dentistry, University of Maryland, School of Dentistry, Baltimore, MD, USA

## Abstract

Mesenchymal stem cells (MSCs) are multipotent cells, which can give rise to variety of cell types, including adipocytes and osteoblasts. Previously, we have shown that cysteine dioxygenase type 1 (*Cdo1*) promoted adipogenesis of primary mouse bone marrow stromal cells (BMSCs) and 3T3-L1 pre-adipocytes via interaction with Pparγ. However, the role of *Cdo1* in osteogenesis remains unclear. Here, we demonstrated that expression of *Cdo1* was elevated during osteoblastic differentiation of BMSCs *in vitro*. Interestingly, knockdown of *Cdo1* by siRNA led to an increased expression of osteogenic related genes, elevated alkaline phosphatase (ALP) activity, and enhanced mineralization. Overexpression of *Cdo1* in BMSCs inversely suppressed the osteogenesis. Furthermore, we found that overexpression of *Cdo1* impaired Wnt signaling and restricted the Wnt3a induced expression of osteogenic transcriptional factors, such as *Runx2* and *Dlx5*. Collectively, our findings indicate *Cdo1* suppresses osteogenic differentiation of BMSCs, through a potential mechanism which involves in Wnt signaling reduction concomitantly.

Mesenchymal stem cells (MSCs) are heterogeneous cell populations with capacity for self-renewal and multipotency of differentiation, which can give rise to multiple cell types, such as adipocytes, chondrocytes, osteocytes, as well as other embryonic lineages^1^,^2^. To date, MSCs are found and isolated from various pre-natal and postnatal tissues, originated from bone marrow^3^, but also umbilical cord blood^4^, adipose tissue^5^, and dental tissues^6^. Further, MSCs are found to play a role in immune-modulation and anti-inflammation at injured sites^7^,^8^. Hence, MSCs have attracted much attention for stem cell-based bone repair^9^,^10^.

The process of osteogenic differentiation of MSCs can be categorized into commitment to osteoprogenitor cells, differentiation into pre-osteoblasts and maturation of osteoblasts^11^,^12^. The mature osteoblasts are capable of synthesizing the bone matrix that eventually becomes mineralized^12^. Mechanistically, the lineage specification of MSCs is a highly controlled process that involves several genetic and epigenetic mechanisms. One of the most extensively studied factors that is important in osteogenesis is runt-related gene 2 (RUNX2), a master transcription factor^13^. And other numerous factors are also required for osteogenesis, including growth factors, hormones, signaling molecules^14^. In addition to osteogenic differentiation, MSC can give rise to adipocytes under suitable conditions. Interestingly, a theoretical inverse relationship has been suggested between osteogenic differentiation and adipogenic differentiation of MSCs^15^,^16^. Several signaling pathways have been investigated to promote osteogenesis and inhibit adipogenesis, such as Wnt signaling, Hedgehog signaling, and NELL-1 signaling^11^,^17^,^18^.

Mammalian cysteine dioxygenase type 1 (*Cdo1*) is an essential enzyme for taurine biosynthesis by catalyzing the oxidation of cysteine to cysteine sulfinic acid^19^. In addition to the enzymatic activity of *Cdo1*, previous studies have also suggested that *Cdo1* expression is upregulated during adipogenesis of human bone marrow-derived MSCs and adipose tissue-derived pre-adipocytes, and *Cdo1* may serve as a marker of adipogenic differentiation of MSCs^20^,^21^. Furthermore, our group have demonstrated that *Cdo1* promoted adipogenic differentiation via interaction with peroxisome proliferator-activated receptor gamma (Pparγ)^21^. Given these findings, and an inverse relationship between osteogenesis and adipogenesis, we hypothesize that *Cdo1* may inhibit osteoblastic differentiation of MSC. To address this hypothesis, in this study, we investigated the expression pattern of *Cdo1* during osteogenic differentiation of BMSCs, and examined the effects of depletion of *Cdo1* and overexpression of *Cdo1* on this osteogenic process. Further, we observed overexpression of *Cdo1* impaired Wnt signaling stimulated by Wnt3a in BMSCs. Our findings indicate *Cdo1* suppresses osteogenesis via inhibition of Wnt signaling.

## Materials and Methods

### Cell Culture

Primary mouse bone marrow stromal cells (BMSCs) were isolated and cultured as described previously^22^. The derived cells were cultured in Dulbecco’s modified Eagle’s medium (DMEM), supplemented with 10% heat-inactivated fetal bovine serum (FBS), 2 mM L-Glutamine, plus 100 U/ml of K-Penicillin G and 100mg/ml of Streptomycin sulfate (all from Gibco) at 37 °C with a humidified atmosphere of 5% CO2. All animal procedures were conducted in accordance with *The Guidelines for the Care and Use* of Laboratory Animals of State Key Laboratory of Oral Diseases, West China Hospital of Stomatology, Sichuan University. To induce osteogenic differentiation, BMSCs were seeded at 5 × 10^3^ cells per well in 24-well-plates, and cultured with osteogenic medium (OS). Osteogenic medium was comprised of 90% α-MEM (Gibco), 10% FBS (Gibco), 100 μM ascorbic acid, 10 mM β-glycerophosphate, and 10nM dexamethasone (all from Sigma).

### Characterization of osteoblastic phenotypes

After several days of osteogenic induction, the cells were fixed in 70% ethanol (Fisher), and alkaline phosphatase (ALP) staining was performed according to the manufacturer’s instructions (System Biosciences). For quantitative determination of ALP activity, 20 μL cell protein solution was incubated with 50 μL ALP stabilizing buffer (Sigma) and 50 μL ALP yellow (pNPP) liquid substrate (sigma) for 20 min at 37 °C. The absorbance was then read on a microplate reader (Bio-Rad) at OD405 nm. Alizarin Red S (ARS) staining was performed to assess the mineralization of extra cellular matrix, after 14 days of osteogenic induction. Briefly, the cells were fixed with 70% ethanol for 1 hour, and stained with 40 mM Alizarin red for 10 min. The stained cultures were destained by 10% CPC, and absorbance of the solution was read at 562 nM.

### Transfection

All *Cdo1*-targetd siRNAs and scramble siRNA (Scr) were purchased from Ribobio (Guangzhou, China).The targeting sequences for siRNA were 5′-AUGCCAAAUUCGAUCAAUAUU-3′ (si1), and 5′-CUGCAAAGGGUGUGUCCUAUU-3′ (si2). BMSCs were overnight plated and transfected with siRNAs using Lipofectamine RNAiMAX reagent (Invitrogen) according to manufacturer’s instructions. For overexpression of *Cdo1*, retroviruses expressing mouse *Cdo1*gene were purchased from Fulengen Inc. (Guangzhou, China).BMSCs were infected with in the presence of polybrene (Sigma) for 24 hr. BMSCs transfected by empty vector were used as control.

### RNA Isolation and Reverse Transcription-PCR (RT-PCR)

Total RNA was isolated using the Trizol reagent (Invitrogen) according to manufacturer’s instructions. Complementary DNA was then synthesized from 2 ug aliquots of RNA using PrimeScript RT Reagent Kit (Takara). Quantitative real-time PCR was performed using SYBR Premix Ex Taq (Takara).The primer sequences used for this analysis were: 5′-ACAACTTTGGCATTGTGGAA-3′ (forward) and 5′-GATGCAGGGATGATGTTCTG -3′ (reverse) for *Gapdh*; 5′-AACCTATGCCCGTTTCCTCTA-3′ (forward) and 5′-GAGTGTAAAGACTTGGTCCACC-3′ (reverse) for *Axin2*; 5′- AATGATTCCATTGGCTTACACCG -3′ (forward) and 5′-GGCATGTATCGAAGGGTGGAC-3′(reverse) for *Cdo1*;5′-GCTCCTCTTAGGGGCCACT-3′ (forward) and 5′-ATTGGGGACCCTTAGGCCAT-3′ (reverse) for *Col1a1*; 5′-CACCACCCGTCTCAGGAATC -3′ (forward) and 5′-GCTTTGCCATAAGAAGCAGAGG-3′(reverse) for *Dlx5*;5′-CAGTGCCACCTTGAACTCAGT -3′ (forward) and 5′-CCGCCCTCATAGAGAACTCC -3′(reverse) for *Dkk1*; 5′-GAAGAGCAAAAAGCGAAACTGG -3′ (forward) and 5′-TTGGCTGCTTGGTGGAATGT-3′(reverse) for *Ibsp*; 5′-GACTGTGGTTACCGTCATGGC-3′ (forward) and 5′-ACTTGGTTTTTCATAACAGCGGA-3′ (reverse) for *Runx2*.

### Western Blot

The BMSCs were lysed with CelLytic MT solution (Sigma), supplemented with protease inhibitor cocktail (Pierce Biotechnology), and centrifuged at 18,000 g for 15 min at 4 °C. Aliquots of the supernatant were subjected to electrophoresis on a 12.5% SDS-PAGE gel. The resolved proteins were then transferred onto nitrocellulose membranes (Bio-Rad). The blots were incubated with primary antibody against *Cdo1* (Abcam), followed by a horseradish peroxidase-conjugated secondary antibody (Boster, Wuhan, China). Antibody-antigen complexes were detected using Luminal/Enhancer Solution and Super Signal West Stable Peroxide Solution (Thermo).

### Luciferase Reporter Assay

One day before transfection, BMSCs were seeded per well into 12-well plate at 10^5^ cells per well. After overnight incubation, the cells were transiently transfected with 1 μg DNA of reporter constructs (TOPflash, Millipore) using 2 μL Lipofectamine 2000^TM^ (Invitrogen) in 50 μL OptiMEM I (Gibco) reduced serum media. Thereafter, the test cells were stimulated with human recombinant Wnt3a (100 ng/ml, System Biosciences); and control cells were treated with phosphate buffered saline (PBS). After 24 hours, cells were lysed and firefly luciferase activity was measured in triplicate according to the manufacturer’s protocol (Promega).The firefly luciferase activity was normalized to protein concentrations.

### Statistical Analysis

Data shown represented as mean ± SD from three independent experiments. Student’s t-test and one-way analysis of variance (ANOVA) were used for single comparisons and multiple comparisons to assess the statistical inference on difference among each pair of data sets, respectively. A *p* value < 0.05 was considered to be statistically significant.

All experimental protocols and procedures were approved by State Key Laboratory of Oral Diseases, West China Hospital of Stomatology, Sichuan University.

## Results

### *Cdo1* is upregulated during osteogenic differentiation of BMSCs

**Figure 1:**
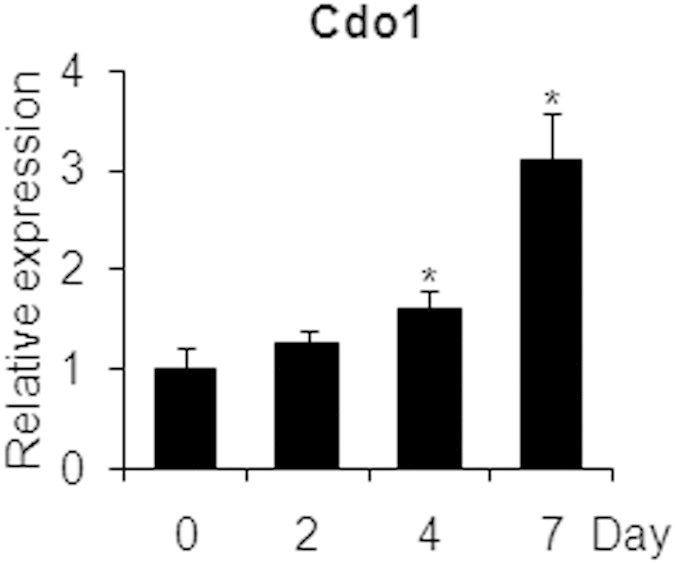
Cysteine dioxygenase type 1 (*Cdo1*) is up-regulated during osteogenic differentiation of mBMSCs. The mRNA expression levels of *Cdo1* during osteogenesis in mBMSCs at 0, 2, 4, 7 days. Asterisks indicate a significant difference compared to the baseline. *p < 0.05.

To explore the role of *Cdo1* in osteogenesis, we first examined the expression level of *Cdo1* during osteogenic differentiation of primary BMSCs. As shown in Fig. 1, the mRNA expression of *Cdo1* was elevated in response to osteogenic stimulation. However, the protein expression of *Cdo1* was detected during osteogenic differentiation of primary BMSCs by Western blot (data not shown).

### Depletion of *Cdo1* enhances osteogenic differentiation of BMSCs

**Figure 2:**
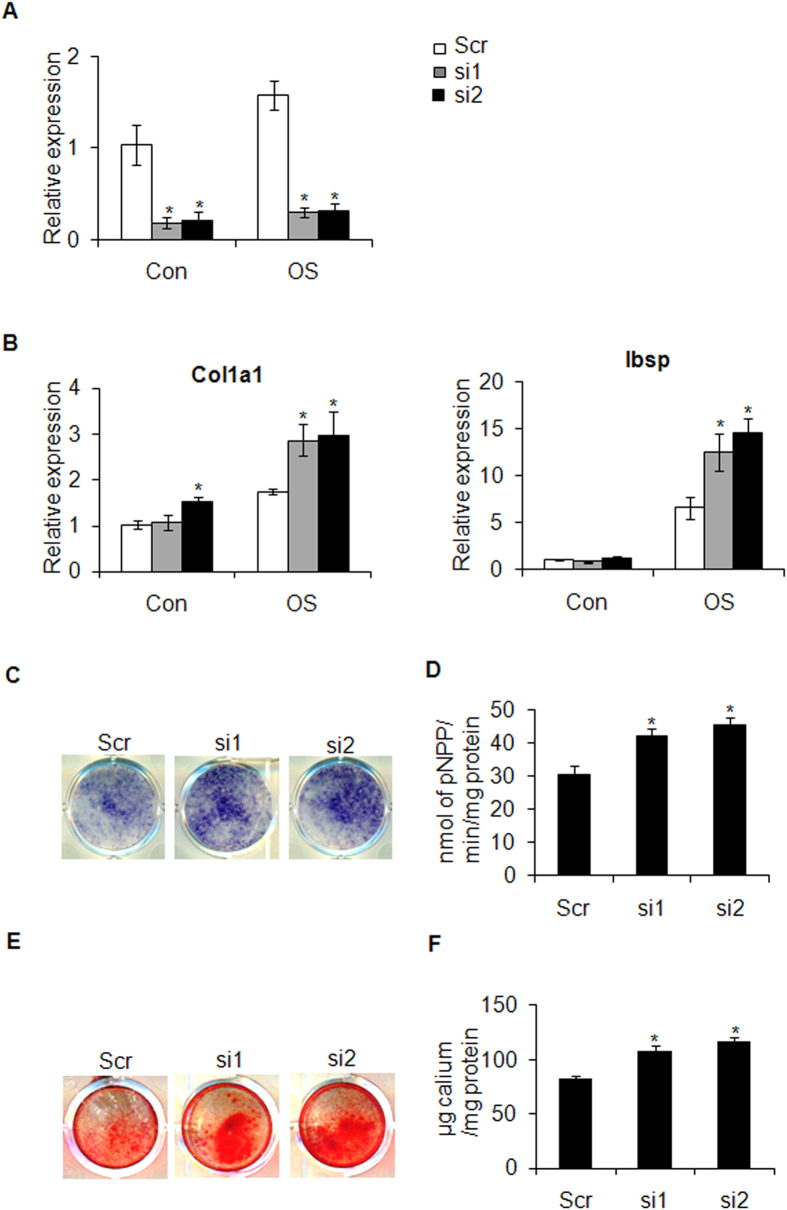
siRNA-mediated depletion of *Cdo1* enhances osteogenic differentiation of mBMSCs. (**A**) The knockdown efficiency of siRNAs targeting *Cdo1*compared to scramble (Scr) siRNA was confirmed by RT-PCR in the presence or absence of osteogenic induction at 3 days after transfection. (**B**) Knockdown of *Cdo1* promoted expression levels of *Col1a1* and *Ibsp* as determined by RT-PCR. (**C**) Knockdown of *Cdo1*enhanced the ALP staining after 7 days of osteogenic induction. (**D**) Knockdown of *Cdo1*enhanced the ALP activity at 3, 7 days of osteogenic induction as determined by quantitative ALP activity assay. (**E**) Knockdown of *Cdo1*enhanced mineralization after 14 days of osteogenic induction. (**F**) Quantification of ARS staining in E. *p < 0.05.

Next, we used two specific siRNAs to knockdown the expression of *Cdo1* in BMSCs, and the knockdown efficiency in the presence or absence of osteogenic stimulus was assessed by RT-PCR (Fig. 2A). After osteogenic induction, we found siRNA-mediated depletion of *Cdo1* significantly promoted expression of osteogenic-related genes, such as *Col1a1* (Collagen, type I, alpha 1), and *Ibsp* (Integrin binding sialoprotein) (Fig. 2B). Consistently, knockdown of *Cdo1* enhanced ALP activity, an early marker of osteoblastic differentiation (Fig. 2C,D). Furthermore, we assessed the extracellular matrix (ECM) mineralization by ARS staining. As shown in Fig. 2E,F, the ECM mineralization was significantly enhanced by depletion of *Cdo1*.

### Overexpression of *Cdo1* inhibits osteogenic differentiation of BMSCs

**Figure 3:**
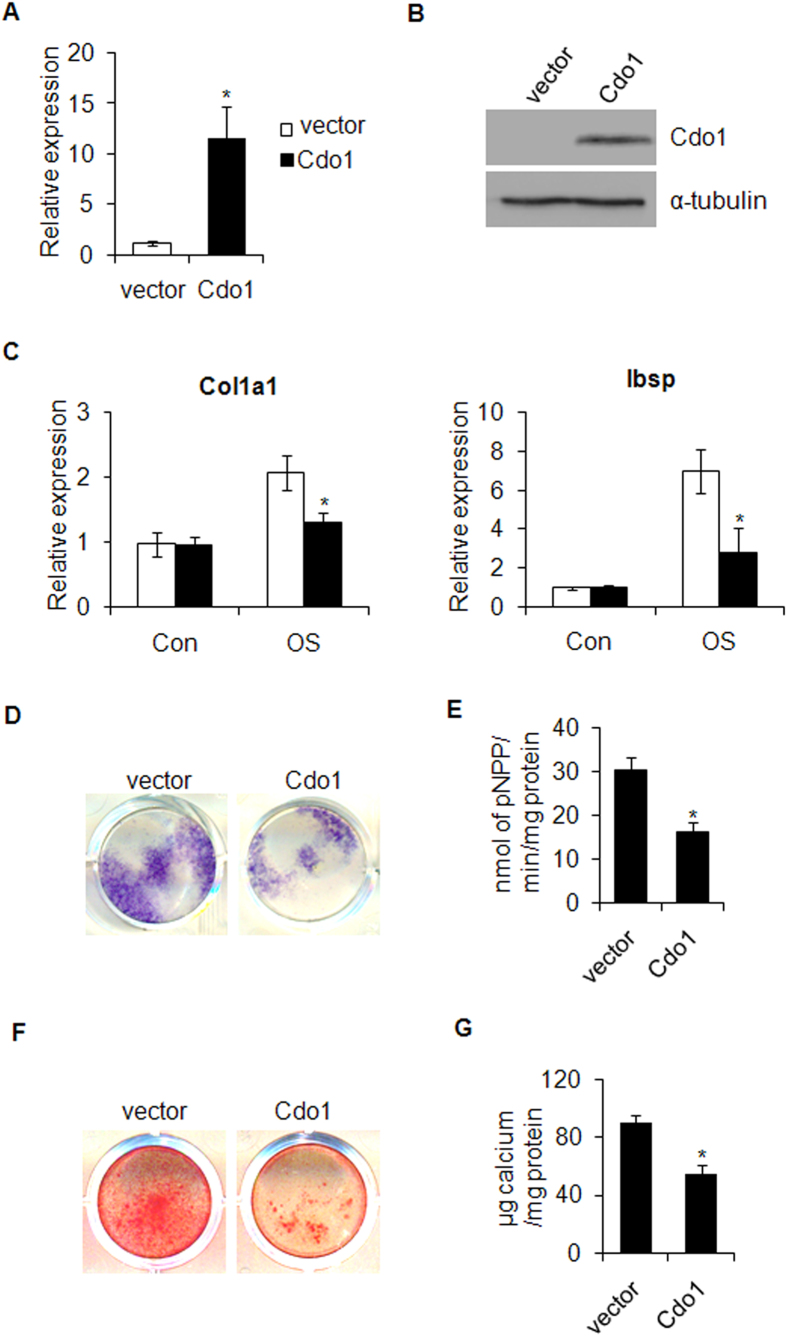
Ectopic overexpression of *Cdo1* suppresses osteogenic differentiation of mBMSCs. (**A**) The overexpression of *Cdo1*in mBMSCs was confirmed by RT-PCR. (**B**) The overexpression of *Cdo1*in mBMSCs was confirmed by Western blot. (**C**) Overexpression of *Cdo1* inhibited mRNA expression levels of *Col1a1* and *Ibsp* stimulated by osteogenic induction. (**D**) Overexpression of *Cdo1*impaired the ALP staining after 7 days of osteogenic induction. (**E**) Overexpression of *Cdo1*inhibited the ALP activity after 7 days of osteogenic induction as determined by quantitative ALP activity assay. (**F**) Overexpression of *Cdo1* reduced mineralization post-14 days of osteogenic induction. (**G**) Quantification of ARS staining in (**F**). *p < 0.05.

To investigate the effects of ectopic overexpression of *Cdo1* on osteogenic differentiation, BMSCs cells were stably transduced with retroviruses expressing *Cdo1* (Fig. 3A,B). As expected, the expression of *Col1a1* and *Ibsp* was downregulated by overexpression of *Cdo1* after osteogenic induction (Fig. 3C). In addition, The ALP activity and ECM mineralization were also impaired by overexpression of *Cdo1* in BMSCs (Fig. 3D–G).

### Overexpression of *Cdo1* impairs Wnt signaling

**Figure 4:**
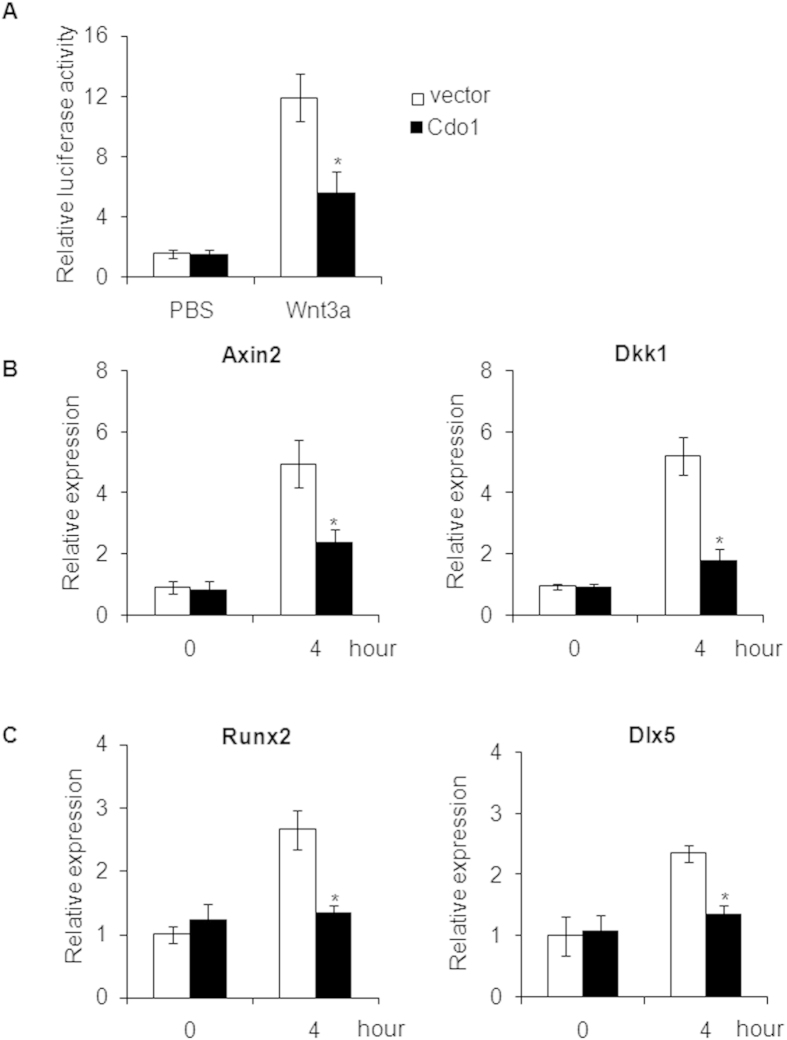
Overexpression of *Cdo1* inhibits Wnt signaling. (**A**) Overexpression of *Cdo1* repressed luciferase activity. (**B**) Overexpression of *Cdo1* inhibited mRNA expression of Wnt target genes, *Axin2* and *Dkk1*, induced by treatment with Wnt3a. (**C**) Overexpression of *Cdo1* inhibited mRNA expression of osteogenic transcription factors, *Runx2* and *Dlx5*, in mBMSCs treated with Wnt3a (100 ng/ml) for 4 hours. *p < 0.05.

Wnt signaling plays an essential role in regulation osteogenic and adipogenic differentiation of MSCs^17^. To investigate whether the inhibitory effect of *Cdo1* on osteogenesis was mediated by Wnt signaling, BMSCs were transfected with TOPflash reporter plasmids. We found that overexpression of *Cdo1* significantly reduced the luciferase activity stimulated by recombinant Wnt3a protein (Fig. 4A). Consistently, the expression of downstream genes, *Axin2* and *Dkk1*, was also downregulated by overexpression of *Cdo1* in response to Wnt3a treatment (Fig. 4B). *Runx2* and *Dlx5* are important transcription factors in osteogenic differentiation of MSCs, and both of them are target genes of Wnt signaling^23^. Further, we found the expression of *Runx2* and *Dlx5* are also inhibited by overexpression of *Cdo1* when treated with Wnt3a. Taken together, *Cdo1* suppresses osteogenic differentiation of BMSCs, through a potential mechanism which involves in Wnt signaling reduction concomitantly.

## Discussion

MSCs have generated a great deal of enthusiasm over the past decade for tissue engineering and regenerative medicine^24^,^25^. Understanding the mechanisms of MSC lineage specification and directing its differentiation in a determined manner are critical for the fundamental and clinical applications^26^,^27^. In the present study, we have found that the expression of *Cdo1* was up-regulated during osteogenic differentiation of BMSCs *in vitro*. While siRNA mediated knockdown of *Cdo1* promoted osteogenic differentiation of BMSCs, ectopic overexpression of *Cdo1* significantly reduced the expression of osteogenic related genes, ALP activity, and ECM mineralization. However, we also noticed that depletion of *Cdo1* did not upregulate ALP activity and ECM mineralization without additional osteogenic stimulus, as shown in Fig. 2c–f. It is possible that depletion of *Cdo1*is not sufficient to initial the osteoblastic commitment of MSCs. To explore the mechanism by which *Cdo1* regulates osteogenesis, we further performed luciferase assay after transfection with TOPflash reporter. And we found that overexpression of *Cdo1* inhibited Wnt signaling, and suppressed expression of Wnt target genes in BMSCs.

Previous studies have suggested that osteogenesis and adipogenesis have an inverse correlation^15^,^28^. We had reported that *Cdo1* promoted adipogenesis, and we further found *Cdo1* inhibited osteogenic differentiation of BMSCs in this study. Although *Cdo1* expression was upregulated in both adipogenesis and osteogenesis, the increased fold of *Cdo1* in adipogenesis was much greater compared to its upregulation in osteogenesis. And the upregulation of *Cdo1* in osteogenesis was observed at medium and late stages of osteogenesis. In contrast, the upregulation of *Cdo1* took place earlier in adipogenesis of mBMSCs^21^. It is possible that a relative higher expression level of *Cdo1* is required to exert its inhibitory effects on osteogenesis. Under physiological conditions, the osteogenic differentiation and adipogenic differentiation of MSCs are well balanced. However, disruption of this homeostasis may lead to bone dysregulations, such as osteoporosis, which is characterized by excessive accumulation of adipocytes and decreased bone mass^29^,^30^. Our results indicate that *Cdo1* may contribute to the development of osteoporosis. While osteoporosis occurs more commonly in aging population, several changes in MSC take place with age, including loss of proliferation potential, decrease in capacity to differentiate into osteoblasts, and increase in capacity to differentiate into adipocytes^31^. It would be interesting to investigate whether *Cdo1* is involved in the age-related changes in MSCs. In addition, our results showed *Cdo1* suppressed the differentiation from MSCs to osteoblasts, but if *Cdo1* affects maturation of osteoblasts still need further investigations.

We also found that overexpression of *Cdo1* impaired Wnt signaling, and inhibited expression of Wnt target genes, such as *Axin2, Dkk1, Runx2*, and *Dlx5*. *Runx2* and *Dlx5* play an important role in initiation of osteogenesis. Thus, our results indicate that such inhibitory effects of *Cdo1* on osteogenesis may be mediated by Wnt signaling. While activation of Wnt signaling promotes osteogenic differentiation of MSCs, it also strongly inhibits adipogenesis, through both-catenin dependent and beta-catenin independent mechanisms^32^,^33^. Further, Song *et al*. reported that loss of Wnt signaling results in a cell-fate shift of preosteoblasts from osteoblasts to adipocytes^34^. It is likely that *Cdo1* is a key factor in lineage specification by regulating Wnt signaling. However, the mechanism by which *Cdo1* regulate wnt signaling still need further investigation. In addition, we have reported that *Cdo1* interacted with Pparγ^21^. Since *Pparγ* can suppress osteogenesis^35^,^36^, activation of *Pparγ* might be an alternative mechanism that *Cdo1* inhibits osteogenesis.

Collectively, we have found that *Cdo1* inhibits osteogenic differentiation by regulating Wnt signaling in primary BMSCs. Together with previous studies, our results indicate that *Cdo1* may play an important role in regulation of the balance between osteogenesis and adipogenesis during MSC differentiation, and upregulation of *Cdo1* may be correlated to the bone-related diseases.

## Additional Information

**How to cite this article**: Zhao, X. *et al*. Cysteine Dioxygenase Type 1 Inhibits Osteogenesis by Regulating Wnt Signaling in Primary Mouse Bone Marrow Stromal Cells. *Sci. Rep*. **6**, 19296; doi: 10.1038/srep19296 (2016).
